# Identifying prognostic biomarkers based on aberrant DNA methylation in kidney renal clear cell carcinoma

**DOI:** 10.18632/oncotarget.14134

**Published:** 2016-12-24

**Authors:** Guang Chen, Yihan Wang, Lu Wang, Wanhai Xu

**Affiliations:** ^1^ Department of Urology, The 4^th^ Affiliated Hospital of Harbin Medical University, Harbin 150001, China; ^2^ College of Bioinformatics Science and Technology, Harbin Medical University, Harbin, 150081, China

**Keywords:** kidney renal clear cell carcinoma, DNA methylation, prognosis, network, gene expression

## Abstract

The outcome of kidney renal clear cell carcinoma (KIRC) differs even among individuals with similar clinical characteristics. DNA methylation is regarded as a regulator of gene expression in cancers, which may be a molecular marker of prognosis. In this study, we aimed to mine novel methylation markers of the prognosis of KIRC. We revealed a total of 2793 genes differentially methylated in their promoter regions (DMGs) and 2979 differentially expressed genes (DEGs) in KIRC tissues compared with normal tissues using The Cancer Genome Atlas datasets. Then, we detected 57 and 34 subpathways enriched among the DMGs and DEGs, respectively, using the R package iSubpathwayMiner. We retained 56 subpathways related to both aberrant methylation and expression based on a hypergeometric test for further analysis. An integrated gene regulatory network was constructed using the regulatory relationships between genes in the subpathways. Using the top 15% of the nodes from the network ranked by degree, survival analysis was performed. We validated four DNA methylation signatures (*RAC2*, *PLCB2*, *VAV1*, and *PARVG*) as being highly correlated with prognosis in KIRC. These findings suggest that DNA methylation might become a prognostic predictor in KIRC and could supplement histological prognostic prediction.

## INTRODUCTION

Roughly 210,000 new cases of renal cell carcinoma, which is the most common malignant tumor derived from the kidney, are diagnosed worldwide each year, accounting for 2–3% of all cancers. At present, kidney renal clear cell carcinoma (KIRC) is the major histological subtype of renal cell carcinoma, accounting for 80–90% of cases [1, 2]. However, the prognosis of KIRC is dire [1]. The most commonly used predictors to assess the risk of patients with KIRC are TNM stage and Fuhrman grade [3, 4]. Nevertheless, patients with similar clinical features or scores may still present variable outcomes. Thus, there is an urgent need to identify new sensitive molecular markers for prognosis and diagnosis, as well as to explore the mechanism in patients with KIRC. One study has found that melanoma cell adhesion molecule (MCAM) and its extracellular matrix interaction partner laminin alpha 4 (LAMA4), which have emerged as the genes most consistently expressed in blood vessels, can predict poor survival in renal cell carcinoma [5]. A five-microRNA signature (hsa-let-7a, hsa-miR-221, hsa-miR-137, hsa-miR-372, and hsa-miR-182) was also shown to be associated with survival and cancer relapse in non-small-cell lung cancer patients [6].

Although cancer initiation and progression are mainly driven by associated genetic alterations, it has emerged that epigenetic changes such as DNA methylation in promoters of tumor-associated genes are extremely important among molecular barriers in neoplastic development [7, 8]. Aberrant DNA methylation in promoter regions is a hallmark of cancer, and it affects gene transcription and genomic integrity in tissue- and time-dependent manners [9–11]. DNA methylation of some genes has already been used as a biological label in the early diagnosis and prognosis of other diseases. For example, CDH1 promoter methylation may be correlated with breast carcinogenesis and associated with poor prognosis in patients with breast cancer [12]. In addition, DNA methylation of the promoter regions of four genes (P16, CDH13, APC, and RUSSF1A) in patients with stage I non-small-cell lung cancer, treated with curative intent by surgery, was shown to be associated with early recurrence [13].

**Figure 1:**
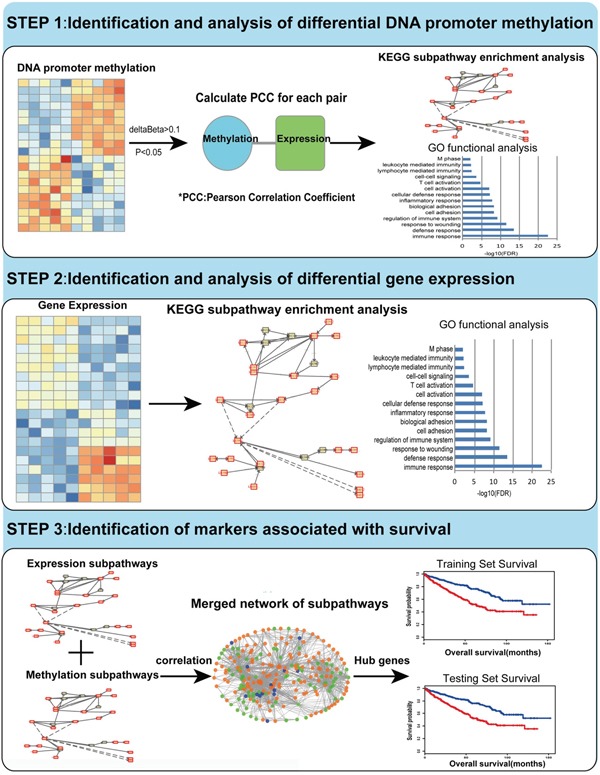
Flow figure indicating study design Step I: We identified and analyzed DMGs through TCGA methylation profiles. By using Pearson Correlation Coefficient method, we got the CpG sites which may influence gene expression. Then gene ontology (GO) functional analysis and KEGG subpathway enrichment analysis were performed for the DMGs. Step II: We identified and analyzed DEGs through mRNA expression profiles. GO functional analysis and KEGG subpathway enrichment analysis were performed for DEGs using the same method mentioned above. Step III: Based on topological property analysis of the integrated network, we identified candidate genes associated with the survival of KIRC both in the training set and testing set.

In this paper, we proposed an integrative framework to predict KIRC patients’ survival (Figure 1). We utilized the DNA methylation profiles and mRNA expression profiles from The Cancer Genome Atlas (TCGA) in this work. For the precise identification of differentially methylated genes (DMGs) and differentially expressed genes (DEGs), we selected 316 samples with both DNA methylation and gene expression profiles for analysis. Then, we used the R package iSubpathwayMiner to detect subpathways enriched among the DMGs and DEGs. We also constructed an integrated gene regulatory network associated with KIRC by the regulatory relationships between genes in the subpathways. Based on topological analysis of this network, we identified 16 hub genes that play crucial roles in patients with KIRC. Finally, we identified and validated four reliable DNA methylation signatures with prognostic utility. Our study not only complements the current prognostic evaluation system of KIRC, but also improves the accuracy of doctors’ prognostic judgments by taking individual heterogeneity into consideration.

## RESULTS

### Identification of KIRC-related DMGs and DEGs at the genome scale

**Figure 2:**
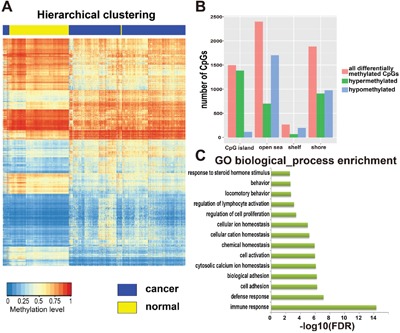
Identification and analysis of differentially methylated genes (DMGs) **A**. Hierarchical clustering of DMGs, rows represent CpG sites and columns represent samples. For a CpG site, red represents higher methylation level, blue represents lower methylation level and white represents medial methylation level for all samples. For a sample, yellow means tumor adjacent tissues and blue means tumor tissues from patients with KIRC. There were 316 cancer samples and 158 adjacent tissues from KIRC patients, and 6050 CpG sites totally. **B**. The distribution of all differentially methylated sites among CpG island, open sea, shelf and shore. **C**. Gene ontology (GO) enrichment analysis of DMGs, the length of green bars represents the P-value of each GO biological process (log10 transformed FDR).

To identify DMGs associated with KIRC, an analysis of genome-wide DNA methylation in gene promoter regions was performed, which involved comparison between 316 cancerous tissues and 158 adjacent tissues from KIRC patients. A total of 194,693 DNA methylation sites were analyzed. To get a precise outcome, the R package ChAMP was utilized to eliminate the batch effect between samples, and probes specific to the sex chromosomes and regions containing single-nucleotide polymorphisms (SNPs) were discarded. Then, based on the linear approximation model, we identified 14,125 differentially methylated sites (p<0.05; Δβ>0.1). Targeting genes whose expression is strongly affected by the DNA methylation level, we evaluated the relationship between DNA methylation level and gene expression level using Pearson's correlation coefficient (PCC). In this way, we identified 6050 CpG sites mapped to 2793 genes. We named these genes DMGs (Figure 2A). Of all the differentially methylated probes, 3060 probes showed hypermethylation in the cancer samples compared with normal samples (51%; [cancer]>[normal]), while 2990 probes showed hypomethylation (49%; [normal]>[cancer]). Moreover, nearly half of the hypermethylated probes were found to be on CpG islands (CGIs, 1384 probes; 45%), while only 5% of the hypomethylated probes were (115 probes; 5%). Almost one-third of hypermethylated probes were on CGI shores (909 probes; 30%), which was similar to the proportion for hypomethylated probes (974 probes; 31%). Only 2% of hypermethylated probes were on CGI shelves (68 probes; 2%), while 7% of hypomethylated probes were on them (199 probes; 7%). Finally, a total of 699 hypermethylated probes were on open sea (699 probes; 23%), while nearly half of the hypomethylated probes were on it (1702 probes; 57%) (Figure 2B). Gene Ontology (GO) functional enrichment performed using the software DAVID revealed that these DMGs are strongly involved in some biological functions highly associated with characteristics of cancers (Figure 2C), such as immune response, cell adhesion, regulation of cell proliferation, and defense response.

**Figure 3:**
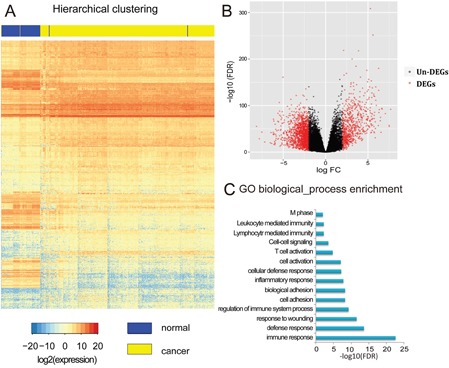
Identification and analysis of differentially expressed genes (DEGs) **A**. Hierarchical clustering of DEGs, rows represent genes and columns represent samples. For a gene, red represents higher expression level, blue represents lower expression level and white represents medial expression level for all samples. For a sample, yellow means tumor adjacent tissues and blue means tumor tissues from patients with KIRC. **B**. Volcano plot of the whole genomic gene expression profile, the red plots represent DEGs with |log_2_FC|>2, FDR<0.01 and black plots represent non-DEGs genes with no significant difference between samples. The abscissa means the value of Fold-Change (log2 transformed FC) and the ordinate means the P-value of differential genes (log10 transformed FDR). **C**. Gene ontology (GO) enrichment analysis of DEGs, the length of blue bars represents the P-value of each GO biological process (log10 transformed FDR).

After eliminating the batch effect of gene expression profiles, a whole-genome gene expression analysis was performed in 316 tumor tissues and 71 normal tissues from patients with KIRC using the R package RUVSeq (see Materials and Methods). The Benjamini–Hochberg (BH) multiple testing method was used to correct the p-values. False discovery rate (FDR) and fold change were used as the criteria (i.e., |log_2_FC|>2, FDR<0.01) for identifying DEGs, which led to 2979 DEGs being assigned for KIRC (Figure 3A). A volcano plot was created to show the values of FDR and logFC for 20,531 genes from the whole genome (Figure 3B). One-third of the total DEGs were overexpressed in cancerous tissue (682 genes, 33%, [cancer]>[normal]), while the others exhibited lower expression (1397 genes, 67%, [normal]>[cancer]). Unsurprisingly, similar functional associations were found by the GO functional enrichment analysis using the software DAVID (Figure 3C), namely, that the DEGs are involved in processes such as cell adhesion, cell proliferation, and immune response. The results indicated that the DNA methylation level may affect gene expression. The associated genes were also found to participate in certain functions that induce KIRC cell proliferation and adhesion.

### Identification of DMG- and DEG-associated subpathways in KIRC

Here, subpathways were applied to investigate biological correlations. The R package iSubpathwayMiner based on the distance similarity method was used to identify subpathways that were significantly enriched among the DMGs and DEGs. For the DNA methylation profiles, 2793 DMGs were enriched in 57 significant subpathways (named DMsubpathways) (p<0.01; Supplementary Table S1). These DMsubpathways are highly associated with the occurrence and progression of cancer, such as the *p53* signaling pathway, *HIF-1* signaling pathway, and calcium signaling pathway. The transcriptional activator HIF1 is the key mediator of the cellular responses to hypoxia and regulates the expression of at least 40 genes that control angiogenesis, invasion, and metastasis of cancer. HIF heterodimers directly induce the expression of Twist by binding to hypoxia response elements (HREs) in the Twist proximal promoter region and promote epithelial-to-mesenchymal transition and a metastatic phenotype [14]. Referred to as a cellular gatekeeper, the *p53* protein acts as a stress-inducing signal to induce antiproliferative cellular responses, such as response to DNA damage, oncogene activation, or hypoxia, in which it subsequently orchestrates biological outcomes including apoptosis, cell cycle arrest, senescence, or the modulation of autophagy [15–18].

DEG-associated subpathways in KIRC were also obtained by the same method. We discovered 34 subpathways enriched for 2979 DEGs (named DEsubpathways) (p<0.01, Supplementary Table S2). Among these 34 DEsubpathways, 28 overlapped with the DMsubpathways (82%), such as the PI3K-Akt signaling pathway, MAPK signaling pathway, NF-kappa B signaling pathway, and cell cycle. Nuclear factor-kappa B is recognized as a critical regulator of immune responses, which could affect cell survival and proliferation, as well as multiple aspects of the immune responses initiated by pattern recognition receptors [19, 20]. A difference between cancer cells and normal cells in the cell cycle module was identified. That it, the cancer group had more cells in the proliferative phase, which is conducive to the immortality of cancer cells.

### Construction of an integrated gene regulatory network based on DMGs and DEGs

**Figure 4:**
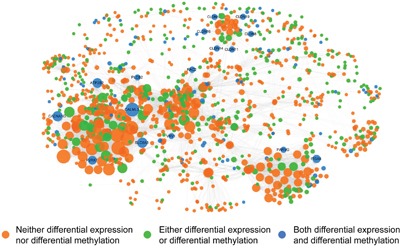
A methylation-associated and expression-related integrated network Orange nodes show the genes are neither DMGs nor DEGs. Green nodes show the genes are either DMGs or DEGs. Blue nodes show the genes are both DMGs and DEGs. The nodes in network represent genes and directed edges mean there is a correlation between genes in at least one subpathway. The nodes size represents its degree.

In the field of oncology, DEGs have been used to identify upstream causal genes through further network analysis, followed by their application for tumor diagnosis and prognosis as new biomarkers. Construction of a specific network is a valid and authentic way of integrating complicated biological correlations and has been applied successfully to identify molecular markers [21, 22]. In our research, to construct an integrated gene regulatory network, we combined subpathways derived from DMGs and DEGs together. For the purpose of finding altered subpathways in both methylation and expression profiles, a hypergeometric test was used to calculate the correlation between each DMsubpathway and each DEsubpathway. Only if there was a strong correlation between one DMsubpathway and one DEsubpathway, the two subpathways were selected for further analysis (hypergeometric test; p<1.0e^−30^). Finally, 56 subpathways under the regulation of both DMGs and DEGs were selected for further analysis (Supplementary Table S3). After extracting the interaction pairs in each reliable subpathway, we constructed a methylation-associated and expression-related integrated gene regulatory network. Visualization of the network was performed using the software Cytoscape (Figure 4). The network contained 1279 nodes and 12,133 edges. The nodes in the network represented genes and directed edges showed that there was a regulatory relationship between two genes in at least one subpathway. The node size reflected its degree in the network. Orange nodes showed that the genes were neither DMGs nor DEGs. Green nodes showed that the genes were either DMGs or DEGs. Blue nodes showed that the genes were both DMGs and DEGs. Finally, after analysis of the topology of this network, 16 genes ranked in the top 15% of the nodes by descending order of degree were chosen. They are not only DMGs but also DEGs. We defined these 16 genes as hub genes, as follows: *CALML3*, *SLC8A3*, *CACNA1G*, *ATP2B2*, *P2RX7*, *ITGA5*, *CLDN8*, *CLDN19*, *CLDN16*, *CLDN14*, *CLDN11*, *CLDN10*, *RAC2*, *PARVG*, *PLCB2*, and *VAV1*.

### Building a survival prediction model based on DNA methylation profile in KIRC

**Figure 5:**
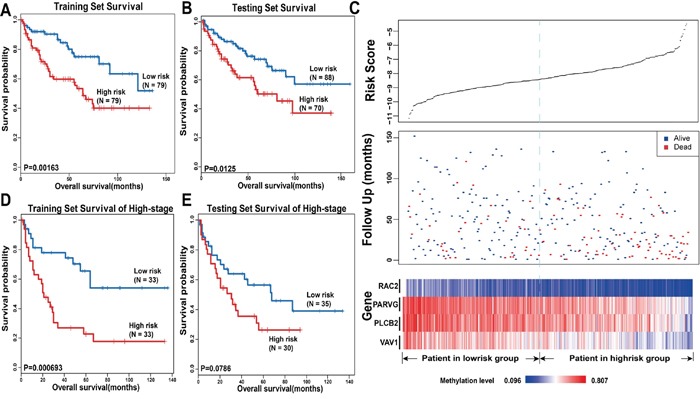
Survival analysis in KIRC The Kaplan-Meier plots show overall survival in high-risk group (red) and low-risk group (blue). The P-value was calculated by log-rank test. Overall survival was indicated in months. **A**. The Kaplan-Meier plots in training set. **B**. The Kaplan-Meier plots in testing set. **C**. The upper panel shows the risk score distribution of all samples that contains both in the training set and testing set. The middle panel shows the status of patients, where red plots represent that patients were dead and blue plots represent patients were alive. The bottom panel is the color-gram of gene methylation value. Rows represent genes and columns represent patients. The black dotted line represents the median methylation signature cutoff dividing patients into high-risk and low-risk groups. **D**. The Kaplan-Meier plots of patients in training set of late stage. **E**. The Kaplan-Meier plots of patients in late stage in testing set.

Next, we used the 16 hub genes to build a KIRC survival prediction model. First, we assigned 316 KIRC samples into a training set (n = 158) and a testing set (n = 158) randomly using criteria such as gender, age, status, and stage with the goal of eliminating the effect of clinical features. Table 1 lists the clinical characteristics of the 316 patients. For each gene, we defined the mean of the methylation level of all the CpG sites within the promoter region as this gene's final DNA methylation value. Next, we used only the training set to test whether these 16 hub genes in KIRC were associated with the survival of patients. Through univariate Cox regression, we found not only that the methylation signature was related to overall survival, but also that the stage and age could affect prognosis. Thus, multivariate Cox regression was applied with the methylation signature, gene methylation, age, and stage as covariates. The results showed that four genes (*RAC2*, *PLCB2*, *VAV1*, *PARVG*) were still associated with overall survival (Table 2, Cox regression, p<0.05). More specifically, we assigned each patient a risk score based on a linear combination of the methylation values of the genes, weighted by the regression coefficients calculated by the aforementioned multivariate Cox regression analysis: Risk score = (−6.401261× *RAC2*) + (−4.704429× *PARVG*) + (−3.03787× *PLCB2*) + (−3.790671× *VAV1*). We divided patients in the training set into high-risk and low-risk groups by using the median of the risk scores as the cut-off point, which was −8.225330351. We used the Kaplan–Meier method to estimate the overall survival times of the patients. Differences between the high-risk and low-risk groups were determined by log-rank test. When using the distribution of risk score to estimate the overall survival of the patients, we found that patients in the high-risk group had a poor survival outcome, and patients with low risk scores had a longer median overall survival time than those with high risk scores (Figure 5A).

**Table 1:** Clinical characteristics of KIRC patients in the training set and testing set

Characteristics	Number of patients			P-value
	All patients n=316	Training set n=158	Testing Set n=158	
State				1^a^
Living	212	106	106	
Dead	104	52	52	
Survival(months)				0.9579^b^
Mean±SD	44.00±35.12	43.90±34.81	44.11±35.54	
Range	1-152	1-136	1-152	
Gender				1^a^
Male	204	102	102	
Female	112	56	56	
Age				0.9208^b^
Mean±SD	63.44±11.86	63.37±11.58	63.51±12.16	
Range	28-92	39-92	28-90	
Stage				1^b^
I	154	77	77	
II	31	15	16	
III	72	36	36	
IV	59	30	29	

^a^p-values were determined using Fisher's exact test.

^b^p-values were determined using Student's t-test.

**Table 2:** Univariate and multivariate survival analysis for KIRC patients in the training set

Variable	Univariate analysis			Multivariate analysis		
	HR(95%CI)	Regression coefficient	p-value	HR(95%CI)	Regression coefficient	p-value
Stage	2.037(1.58-2.625)	0.711	3.9×10^−8^			
Age	1.03(1.007-1.053)	0.030	1.1×10^−2^			
Gender	1.117(0.6255-1.995)	0.111	7.1×10^−1^			
RAC2	6.73×10^−4^ (8.676e-06-0.05224)	−7.303	1.0×10^−3^	1.66×10^−3^ (1.718e-05-0.1603)	−6.401	6.05×10^−3^
PARVG	4.27×10^−3^ (0.0002186-0.08337)	−5.456	3.2×10^−4^	9.06×10^−3^ (0.0002744-0.2988)	−4.704	8.36×10^−3^
PLCB2	6.02×10^−3^ (0.0005959-0.06077)	−5.113	1.47×10^−5^	0.04794(0.003973-0.5784)	−3.038	1.68×10^−2^
VAV1	4.99×10^−3^ (0.0002597-0.09591)	−5.300	4.4×10^−4^	0.02258(0.001371-0.372)	−3.791	8.01×10^−3^

Next, patients in the testing set were utilized to validate the predictive prognostic ability of the four significant methylation signatures. We calculated the four-methylation signature risk score with the same risk score formula as acquired from the training set for each of the 158 patients in the testing set; we then classified the patients into the high-risk or low-risk group based on the same cut-off point as used in the training set. The Kaplan–Meier method and log-rank test were used to examine the significance of the difference between the two subgroups in the testing set. This analysis revealed a significant decrease in the survival of patients whose risk scores were high in the testing set (log-rank test, p=0.0125, Figure 5B). Patients in the low-risk group had longer median survival than those in the high-risk group (45 months versus 26.5 months). The Figure 5C shows the risk scores, follow-up status, and gene methylation signatures of 316 patients in both the training set and the testing set. Compared with patients in the low-risk group, high-risk patients tended to have a lower methylation level for the four genes. More deaths occurred in patients with high risk scores than in those with low ones (Figure 5C).

To evaluate whether the four prognostic predictors also reflect specific clinical characteristics, we compared the clinical characteristics between the high-risk group and the low-risk group and found a marked difference in TNM stage (chi-square test, T: p=0.004938; N: p=0.05684; M: p=0.03502), indicating that, compared with the low-risk group, tumors of the patients in the high-risk group tended to be larger in size, to invade adjacent surrounding tissues more, and to metastasize to lymph nodes and distant organs. Moreover, the differences in pathology grade and stage were also significant between the two groups (grade: p=0.0006263, stage: p=0.003686, chi-square test), indicating that tumors of the patients in the low-risk group were inclined to be well differentiated and to have a lower clinical stage. Furthermore, we collected 66 patients and 65 patients at a higher stage (stage III/IV) among all of the samples from the training set and the testing set, respectively, and constructed new training and testing sets corresponding to those in the previous analysis. Then, we performed survival analysis to investigate whether the four methylation signatures could distinguish high-risk patients from low-risk ones with a higher stage. By Cox regression, the four methylation signatures were found to be related to overall survival. As shown in Figure 5D, there was a significant difference between the survival time of the high-risk group and that of the low-risk group in the new training set. In the new testing set, the curves were separated perfectly, but the P-value was slightly above the 5% level. This may because the sample size was too small to reach significance (Figure 5E). In conclusion, the four DNA methylation signatures can predict overall survival of KIRC patients successfully, and can predict metastasis and the malignancy of tumors, which may have prognostic and therapeutic implications for those involved in decision-making regarding the treatment of these patients.

## DISCUSSION

KIRC is the eighth most common cancer with the highest fatality rate of all genitourinary tumors, with approximately 65,000 new cases and approximately 13,000 deaths annually in the United States [23–25]. Thus, there is an urgent need to identify reliable molecular markers to predict patient survival in KIRC. Although a few markers were revealed to be related to the prognosis of KIRC in previous studies, the results were not consistent. There are a range of possible reasons for this inconsistency, such as the ethnicity of the subjects, the analytical methods selected, and the number of patients included. Given this background, more accurate and reliable results can be obtained by using a scientific approach to identify molecular markers for the prognosis of KIRC.

In this study, we systematically analyzed KIRC genomic data, including DNA promoter methylation and gene expression, to discover novel and reliable molecular markers. First, we eliminated factors such as the batch effect that can influence outcomes by using R packages. Second, to better understand the effect of DNA promoter methylation on gene expression, we selected 316 samples with both DNA promoter methylation profiles and mRNA expression profiles, so the individual personal factors were minimized. We also calculated the PCC between each differentially methylated DNA promoter site and the matched gene expression data in the same individual. The identified DEGs not only confirmed some previous research findings, but also provided new findings. For example, TNFAIP, a well-known tumor α-induced protein that acts as a natural brake on inflammation, was found to be upregulated in this KIRC research [26]. Another DEG, SLC6A3, which has already been implicated in lung and breast cancer, was here found to be involved in KIRC for the first time [27, 28].

Cancer is extraordinarily complex in that its emergence involves a multigene process that contributes to malignant transformation. Thus, it is necessary to construct a biological network to shed light on the initiation and progression of cancer. Although a few studies have constructed biological networks, most of them only constructed networks based on a single genomic profile. However, in this study, we performed enrichment analysis based on both DNA promoter methylation profile and gene expression profile at the same time. Biological subpathways instead of pathways enriched for DEGs were identified, which can increase the validity of the enrichment analysis. This is because the subpathway enrichment analysis method, with the ability to identify unregulated local areas, takes complicated structural information into account, and can reveal the correlation between diseases and biological pathways more precisely. After the enrichment analysis, we analyzed the relationship between each DMsubpathway and DEsubpathway by a hypergeometric test. The results showed there were 56 subpathways shared by both profiles, such as the HIF-1 signaling pathway and the calcium signaling pathway. With the goal of identifying markers that have a strong relationship with KIRC, we built an integrated gene regulatory network. Hub genes were typically defined as the top 15% of the nodes ranked by degree [29–31], including about 190 genes. In this case, as selection for further analysis, we required the nodes to be DMGs overlapping with DEGs; using this criterion, only 16 hub genes were selected. Since the hub genes play a central role in the biological network, they were considered as candidate signatures for further study.

Using Cox regression analysis, DNA promoter methylation of four methylation signatures (*RAC2*, *PLCB2*, *VAV1*, *PARVG*) was found to be associated with survival in both the training set and the testing set. Specifically, after eliminating other characteristics such as age or stage, patients with high-risk scores were found to have poor survival compared with the other patients with low-risk scores; furthermore, patients in the high-risk group showed the features of invasion, metastasis, and a poor pathology grade. The four methylation signatures thus have potential as molecular markers to predict patient prognosis in a clinical context, for the following two reasons: (1) The four methylation signatures can predict disease status, progression, and patient survival in a precise way, as mentioned above. (2) Although a few studies have analyzed genomic profiles in KIRC and identified their potential clinical relevance [32–35], these signatures have limited usefulness in clinical practice because of the large numbers of genes included. However, in this study, we used the DNA methylation level of only four highly prognostic DNA promoter regions; this low number of markers makes our classifier faster to use and more feasible in a clinical context.

The final four methylation signatures participate in the regulation of tumor cell function, which further demonstrates the validity of our work. For example, the guanine nucleotide exchange factor *VAV1*, which is an activator of Rho family GTPases, is unregulated in many pancreatic cancers, where it facilitates the survival and migration of tumor cells [36, 37]. *RAC2* controls macrophage differentiation from M1 to M2, which is well known to be important in tumor progression and the metastatic phenotype. In addition, a long noncoding RNA was shown to indicate a poor prognosis of hepatocellular carcinoma via upregulation of RhoA/Rac2 signaling. Moreover, Rac2 was shown to be associated with a poor prognosis in patients with systemic mastocytosis and acute myeloid leukemia. Thus, we believe RAC2 has a significant effect in tumor growth, angiogenesis, and metastasis [38–40]. PLC in breast cancer has also been demonstrated to be overexpressed compared with the level in normal tissue. This suggests that upregulation of PLC-γ1 is associated with growth factor-mediated tumor cell migration and invasiveness [41–43]. Furthermore, PARVG, located on 22q13, was identified as a candidate tumor suppressor gene for colorectal and breast cancer, and ILK-γ-parvin complex was revealed to be critically involved in the initial integrin signaling for leukocyte migration [44, 45]. A study also showed that Vav1 plays a unique role in T-cell leukemia survival by selectively triggering the Rac2-Akt axis and elevating the expression of anti-apoptotic Bcl-2. All of these findings suggest that our biomarkers can be linked together as a pathway associated with survival [46].

Owing to individual differences, the current clinical prognostic system cannot precisely predict each patient's survival and patients with similar clinical features may have diverse outcomes. Thus, there is a demand to increase the prognostic value for the current staging system. Our study suggests that, if we add the four DNA methylation signatures to the current clinical prognostic system, it may be easier for doctors to predict the survival of patients with similar clinical features or the rate of metastasis and to improve the outcome by establishing a better therapy for patients in the high-risk group.

## MATERIALS AND METHODS

### Retrieval of data on DNA methylation in the gene promoter regions and gene expression profiles

Data on DNA methylation in the gene promoter regions and gene expression data of KIRC were collected from The Cancer Genome Atlas (https://tcga-data.nci.nih.gov/tcga/). Two sets of paired data (cancerous and normal adjacent tissues from KIRC patients) were downloaded, including mRNA expression profiles (level 3 data, RNA-seq Version 2, Illumina) of 532 cancer samples and 71 adjacent tissues from KIRC patients; the gene set type is Refseq, DNA methylation profiles (level 3 data, Infinium HumanMethylation450BeadChIP) of 321 cancer samples and 158 adjacent tissues from KIRC patients. The clinical data included retrospectively identified information of 532 patients, such as gender, age, and clinical status.

### Identification of DMGs associated with KIRC

The R package ChAMP [47] was used to identify DMGs among the DNA methylation profiles in gene promoter regions. This study only considered CpG sites around DNA promoter regions [2000 bp upstream of the transcription start site (TSS) to 500 bp downstream of the TSS]. CGIs are defined as regions with CG content >50% and length >200 bp, and CGI shores are regions flanking CGIs in their upstream 2-kb region. CGI shelves are regions up to 2 kb from CGI shores and beyond of these are open sea. After discarding probes specific for the sex chromosomes or regions containing SNPs, there were 194,693 eligible sites for further analysis. To minimize the batch effects between samples, we used ComBat to process the data. Based on the linear model of the R package limma, we identified differentially methylated sites. The BH method was used to adjust the P-value of the model. The threshold for defining a differentially methylated site was that the adjusted P-value must be less than 0.05, and the differential level (Δβ value) between cancer and normal tissues must be greater than 0.1. PCC was calculated to assess the correlation between DNA methylation values of differentially methylated CpG sites and the corresponding mRNA expression values. Only when the P-value was less than 0.05 and there was a negative correlation between the methylation and expression did we use genes for further analysis. We named the genes mapped by the differentially methylated sites as DMGs.

### Identification of DEGs associated with KIRC

The R package RUVSeq [44] was used to minimize batch effects between cancer and normal tissues, and based on the edgeR [48] algorithm we identified DEGs with mRNA expression profiles. The BH method was used to adjust the P-value (FDR). The thresholds were FDR<0.01 and |log_2_FC|>2. We named the genes that satisfied these criterias as DEGs.

### GO and subpathway enrichment analyses

Based on DMGs and DEGs, DAVID was used for GO enrichment analysis [49]. Fisher's exact test with multiple test correction (FDR<0.05) was used to obtain significant GO terms associated with KIRC. We also used the R package iSubpathwayMiner [50] to identify subpathways enriched among DEGs. First, we used the package iSubpathwayMiner to convert each complex structure of pathways from KEGG to a simple directed graph with the genes as nodes. Two nodes in a directed graph were connected by an edge if there was a reaction between them. Then, based on distance similarity among genes, we identified subpathways associated with KIRC. Compared with the methods used to identify entire pathways, our method can identify the subpathways more precisely because the results are sometimes highly significant in our subpathway identification, but not significant in the entire pathway identification. Thus, this approach can identify local disordered regions of entire pathways, which makes further research reliable.

### Constructing an integrated network associated with KIRC

Hypergeometric tests were used to assess the correlation between subpathways of methylation profiles and subpathways of expression profiles. Only when the P-values were less than 1.0e^−30^ were these subpathways selected for further analysis. This means that the remaining subpathways were involved in both DNA methylation and gene expression. After extracting the correlation pairs, the software Cytoscape [51] was used to visualize the network, in which nodes represent genes and edges represent relationships between genes in at least one subpathway. The topological features of the network provide a quantified method to describe networks. In this research, we used the most common topological features of a network, the degree, which can represent the number of neighboring nodes, or the number of edges linked to the node.

### Survival analysis

To identify and validate prognostic markers with target genes, the 316 samples were randomly assigned to a training set (n=158) or a testing set (n=158). The two sample sets were required similar clinical features such as stage or gender. We used the term hub genes to refer to the top 15% of the nodes in the network ranked by descending order of degree, which would be not only DMGs but also DEGs. Then, we used univariate Cox regression analysis to assess the association between survival and DNA methylation levels of hub genes as well as other clinical factors. As the clinical features were also related to patient survival, multivariate Cox regression analysis was used to assess the independent contribution of each gene to prognosis, with the gene methylation, age, gender, and stage as covariates. A regression coefficient with a plus sign indicates that increased methylation is associated with an increased risk of mortality (risk genes) and a minus sign indicates that increased methylation is associated with a reduced risk of mortality (protective genes). After selecting hub genes that were significantly associated with survival (p<0.05), according to a linear combination of methylation levels of genes, a mathematical formula for survival prediction was constructed. Specifically, the risk score formula for each patient was calculated as follows:

$$\text{Risk Score}=\;\sum_{i=1}^{n}\beta_{i}X_{i}$$

where β_i_ is the Cox regression coefficient of hub gene i in the training set, X_i_ is the methylation level of hub gene i, and n is the number of hub genes that are significantly associated with survival. Thus, all patients in the training set were dichotomized into high-risk and low-risk groups using the median risk score as the cut-off point. To estimate overall survival, the Kaplan–Meier method was used and the log-rank test was used to determine whether there was a significant difference in survival between the two risk groups. Then, the testing set was used to validate the four methylation signatures. The regression coefficients and the threshold of risk score derived from the training set were directly applied to the methylation profiles of the testing set, and then the patients in the testing set were divided into high-risk and low-risk groups. Evaluation of the survival time and comparison between two groups were performed in the same way as for the training set.

## SUPPLEMENTARY TABLES








