# Supplementary material for: Identifying prognostic biomarkers based on aberrant DNA methylation in kidney renal clear cell carcinoma
Source: Oncotarget. 2016 Dec 24;8(3):5268–80. doi: 10.18632/oncotarget.14134 (PMC5354907; doi:10.18632/oncotarget.14134)
Supplement: Supplementary file 1 [file oncotarget-08-5268-s001.pdf]

Identifying prognostic biomarkers based on aberrant DNA methylation in kidney renal clear cell carcinoma

SUPPLEMENTARY TABLES

Supplementary Table S1: Subpathways enriched by differentially methylated genes

See Supplementary File 1

Supplementary Table S2: Subpathways enriched by differentially expressed genes

See Supplementary File 2

Supplementary Table S3: subpathways under the regulation of both DMGs and DEGs

See Supplementary File 3
